# Zampanolide, a Microtubule-Stabilizing Agent, Is Active in Resistant Cancer Cells and Inhibits Cell Migration

**DOI:** 10.3390/ijms18050971

**Published:** 2017-05-03

**Authors:** Jessica J. Field, Peter T. Northcote, Ian Paterson, Karl-Heinz Altmann, J. Fernando Díaz, John H. Miller

**Affiliations:** 1 Centre for Biodiscovery and Schools, Victoria University of Wellington, PO Box 600, Wellington 6140, New Zealand; jessica.field.nz@gmail.com (J.J.F.); peter.northcote@vuw.ac.nz (P.T.N.); 2 Biological Sciences, Victoria University of Wellington, PO Box 600, Wellington 6140, New Zealand; 3 Chemical and Physical Sciences, Victoria University of Wellington, PO Box 600, Wellington 6140, New Zealand; 4 Department of Chemistry, Cambridge University, Cambridge CB2 1EW, UK; ip100@cam.ac.uk; 5 Department of Chemistry and Applied Biosciences, Swiss Federal Institute of Technology (ETH), Zürich 8093, Switzerland; karl-heinz.altmann@pharma.ethz.ch; 6 Centro de Investigaciones Biológicas (CIB), CSIC, Madrid 28040, Spain; fer@cib.csic.es

**Keywords:** anticancer, cell migration, discodermolide, ixabepilone, microtubule, paclitaxel, zampanolide

## Abstract

Zampanolide, first discovered in a sponge extract in 1996 and later identified as a microtubule-stabilizing agent in 2009, is a covalent binding secondary metabolite with potent, low nanomolar activity in mammalian cells. Zampanolide was not susceptible to single amino acid mutations at the taxoid site of β-tubulin in human ovarian cancer 1A9 cells, despite evidence that it selectively binds to the taxoid site. As expected, it did not synergize with other taxoid site microtubule-stabilizing agents (paclitaxel, ixabepilone, discodermolide), but surprisingly also did not synergize in 1A9 cells with laulimalide/peloruside binding site agents either. Efforts to generate a zampanolide-resistant cell line were unsuccessful. Using a standard wound scratch assay in cell culture, it was an effective inhibitor of migration of human umbilical vein endothelial cells (HUVEC) and fibroblast cells (D551). These properties of covalent binding, the ability to inhibit cell growth in paclitaxel and epothilone resistant cells, and the ability to inhibit cell migration suggest that it would be of interest to investigate zampanolide in preclinical animal models to determine if it is effective in vivo at preventing tumor growth and metastasis.

## 1. Introduction

Zampanolide (ZMP) is a marine sponge secondary metabolite that stabilizes microtubules (MTs), arrests cells in mitosis, and inhibits cell proliferation in the low nanomolar range [1]. ZMP binds covalently to its primary target β-tubulin [2], similar to two other microtubule-stabilizing agents (MSAs) cyclostreptin [3] and taccalonolide AJ [4]. Because of its covalent binding [5], ZMP may evade multi-drug resistance that results from overexpression of the *p*-glycoprotein (P-gp) drug efflux pump, since any ZMP that covalently binds is no longer available to interact with the drug efflux pumps [5]. Previous work in the A2780AD human ovarian carcinoma cell line showed that the resistance ratio was 1.4 for ZMP and 208 for paclitaxel (PTX) [1]. Resistance ratio = (IC_50_ in resistant cells)/(IC_50_ in parental cells). A2780AD cells overexpress the P-gp drug efflux pump. Cell resistance to drugs can also arise, however, as a result of changes in β-tubulin isotype expression and mutations in the tubulin gene, particularly those mutations that affect the binding pocket of an MSA. Cabral et al. [6] were the first to show resistance to PTX resulting from a tubulin mutation induced by irradiation, in this case a temperature-sensitive mutation in α-tubulin that conferred 2–3-fold resistance to PTX in Chinese hamster ovary (CHO) cells. Schibler and Cabral [7] later showed in CHO cells that 59 out of 139 PTX-resistant mutants displayed absolute requirements for PTX, presumably due to unstable MTs, and 13 of these mutants had point mutations in α- and β-tubulin. Yin et al. [8] later generated β1-tubulin mutants by site-directed mutagenesis and found that three mutations, Ala187Thr, Ala250Val, and Arg308Cys, caused PTX and epothilone resistance, as well as increased sensitivity to MT-destabilizing agents. Giannakakou et al. [9,10], using ovarian carcinoma 1A9 cells, investigated the effect of different point mutations induced by the selection in high concentrations of the MSAs PTX and epothilone. Single point mutations were found in β1-tubulin that led to resistance to one or the other MSA, or to both. Later studies by Kanakkanthara et al. [11] and Begaye et al. [12] described the effect of similar mutations at the laulimalide/peloruside binding site. Arguments for and against point mutations significantly contributing to cancer cell resistance in the clinic exist; however, it is now generally accepted that mutations can lead to the resistance of tumor cells in patients [8,13,14].

One area of interest in MSA anticancer chemotherapy is the ability of two drugs, when given in combination, to synergize together and have a greater than additive effect on growth inhibition than when given alone. The delivery of two drugs also reduces the likelihood that a cancer cell can acquire resistance to one of the two drugs when the other is still present to prevent survival of the cell. Two binding sites for MSAs have been identified, the taxoid site on β-tubulin that faces the inside of the assembled MT [15] and the laulimalide/peloruside site on the external face of the assembled MT [16]. The methods of stabilization differ between these two sites [15,16]; hence, PTX synergizes with peloruside or laulimalide, but not with epothilone or docetaxel [17,18,19]. Discodermolide, another taxoid site MSA, synergizes with PTX, presumably because the two MSAs occupy distinct positions in the taxoid binding pocket and have different mechanisms of stabilization [20,21,22,23]. Discodermolide has recently been shown to stabilize the M-loop of tubulin in a different way to PTX, and this at least partially explains the synergy between the two taxoid site ligands [24]. Clinical interest is strong on combination therapy between MSAs; however, most combination trials in the literature that involve MSAs are in combination with other classes of anti-cancer drugs that do not target the MT.

Metastasis of cancer cells from a primary tumor to other sites in the body is a major, and lethal, problem in cancer. Metastasis involves a complex cascade of events, beginning with the formation of a tumor blood supply, followed by escape of tumor cells from the primary tumor mass, invasion and migration through the extracellular matrix/basal lamina utilizing heparanase and matrix metalloproteinase enzymes, entry into the tumor blood supply, and later extravasation at a distant site to form secondary tumor foci [25]. Cell migratory ability is an important component of the process and is also necessary for angiogenesis and tumor vascularization [26]. Metastatic ability can be monitored in culture by measuring cell migration using methods such as the wound scratch assay [27], Boyden chambers to measure transmembrane migration and invasion [28], as well as binding to and migration into a collagenous basement membrane matrix such as Matrigel™ that models the extracellular matrix of cells [29]. MSAs have been shown to inhibit cell migration in culture, a property that may enhance their overall inhibitory effect on cancer cell proliferation and improve patient survival [30,31,32,33]. The inhibition of MT dynamics and cytoskeletal regulatory molecules such as Rho-GTPases have been shown to play a role in cell movement over a substratum [34,35]. Ganguly et al. [36,37] directly investigated the effects of MT-targeting drugs on cell migration and showed that their effects on cell movement occur at concentrations lower than those required to block cell proliferation or to alter tubulin polymer formation. The MTs at the leading edge of the cell are more static than the dynamic MTs at the trailing edge of the cell. The latter allow reorganisation and remodeling of the MT skeleton. Blocking MT dynamicity does not stop the movement of cells but makes it more random, since MTs restrain cell movement by controlling the trailing portion of the migrating cell. Treatment with a MT-targeting drug prevents tail retraction, but lamillipodia extension still occurs, and the cell can still elongate.

**Figure 1:**
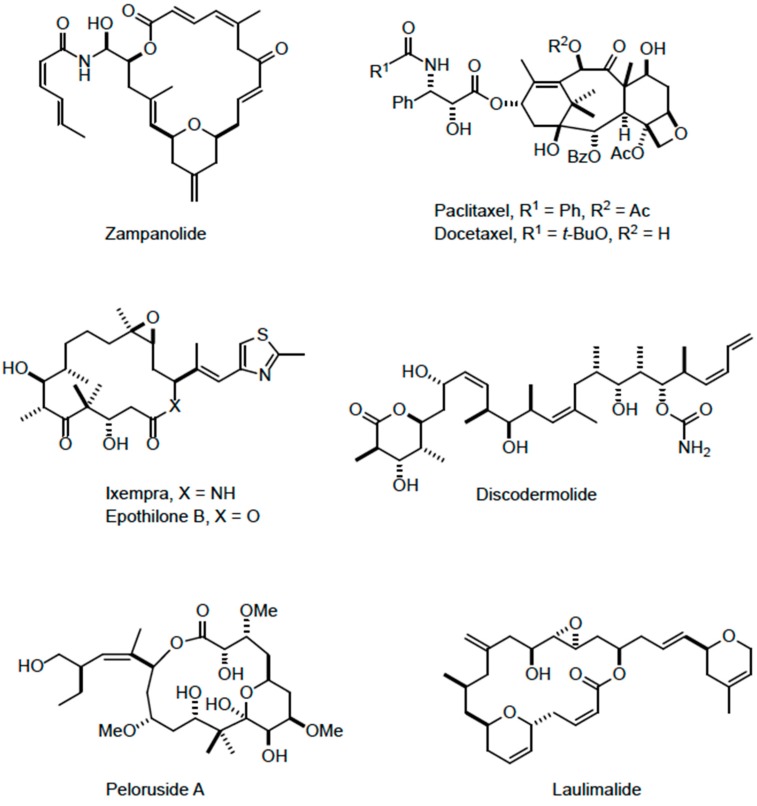
Structure of the compounds.

The aim of the present study was to further characterize the action of ZMP in cultured cells, comparing its activity to other MSAs, both taxoid site and laulimalide/peloruside site binding agents. The MSAs investigated included the taxoid site ligands, ZMP, PTX, (Taxol^®^ Bristol-Myers Squibb), docetaxel (Taxotere^®^, Rhone Poulenc Rorer), ixabepilone (Ixempra^®^, Bristol-Myers Squibb), and discodermolide, and the laulimalide/peloruside site ligands, laulimalide and peloruside A (see Figure 1 for structures of the compounds). The ability of ZMP to remain active in tubulin mutant cell lines was investigated. In addition, the ability of ZMP to synergize with other MSAs was tested, and its effects on cell migration in a wound scratch assay were determined.

## 2. Results

### 2.1. Growth Inhibition by Zampanolide and Other Microtubule-Stabilizing Agents in Different Cell Lines

Human 1A9 ovarian carcinoma cells were treated with MSAs, including ZMP, three other taxoid site MSAs, and two laulimalide/peloruside site MSAs, and the IC_50_ values for inhibition of proliferation were calculated (Table 1). The IC_50_ values ranged from 3.6 to 23.3 nM. ZMP was then tested in other cell lines to determine the consistency of its action and to compare natural ZMP isolated and purified from a marine sponge [1] with chemically synthesized ZMP [38] (Table 2). Although the IC_50_ values varied to some extent between different cell lines, there was no major difference between the natural ZMP and the synthetic ZMP.

### 2.2. Action of Zampanolide on Cells with β-Tubulin Mutations

**Figure 2:**
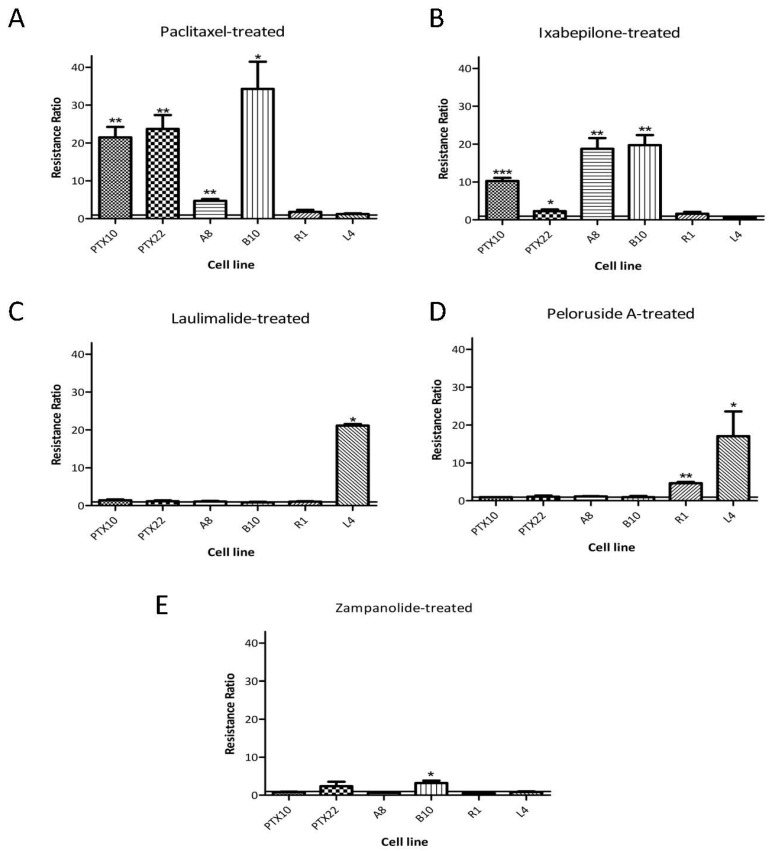
Resistance ratios of MSAs in β-tubulin mutant cell lines. β-Tubulin mutant cell lines and the parental 1A9 cell line were treated with serial dilutions of MSAs for 3 days, and the IC_50_ values were calculated. Resistance ratios (mutant cell IC_50_/parental cell IC_50_) for (**A**) Paclitaxel; (**B**) Ixabepilone; (**C**) Laulimalide; (**D**) Peloruside A, and (**E**) zampanolide are presented as the mean ± SEM, *n* ≥ 3 independent experiments. The specific IC_50_ values are included in Table 3. A one-sample Student’s *t*-test was carried out to determine if the resistance ratios were significantly different from 1.0 (* *p* < 0.05; ** *p* < 0.01; *** *p* < 0.001).

The effect of mutant tubulins on the activity of ZMP was investigated using a collection of 1A9 cell lines that were generated by treatment for extended periods of time to step-wise increases in an MSA, resulting in single amino acid mutations in β1-tubulin [9,10,11]. The spontaneous, stable mutations were either located at the taxoid site or at the laulimalide/peloruside site on tubulin (Table 3). The resistance ratios (IC_50_ mutant/IC_50_ parent) are graphed in Figure 2, and the IC_50_ values are presented in Table 3. The actual values for the resistance ratios are presented in Supplementary Data Table S1. There was some crossover in the specificity of the mutations generated by high concentrations of PTX or epothilone A, with the PTX10 and A8 cell lines being resistant to both PTX and ixabepilone. B10, the mutant cell line generated by high concentrations of epothilone B, also showed significant crossover with both PTX and ixabepilone showing reduced potency in that cell line. A similar crossover was seen for the 1A9-L4 cell line generated in the presence of high concentrations of laulimalide which was resistant to both laulimalide and peloruside. None of the mutant taxoid site cell lines showed any major resistance to zampanolide, although the resistance ratio for PTX22 was 2.4 ± 0.2 (*p*-value just greater than the cut-off for significance of *p* < 0.05) and the resistance ratio for B10 was 3.2 ± 0.6 (*p* < 0.02).

An attempt was made to generate a ZMP-resistant cell line by culturing 1A9 cells for approximately one year in gradually increasing concentrations of ZMP, similar to the procedure used to generate the PTX-, epothilone-, peloruside-, and laulimalide-resistant 1A9 cell lines. The pretreatment with ZMP, however, failed to generate a ZMP-resistant cell line and actually led to a cell line that was slightly more sensitive to ZMP (resistance ratio of 0.59). Despite not being resistant to ZMP, the cells acquired significant resistance to PTX (resistance ratio of 11.2), suggesting a mutation in β-tubulin at or near the taxoid site. However, there was no resistance to ixabepilone (resistance ratio 0.49), nor to peloruside A and laulimalide (resistance ratios of 0.66 and 0.40, respectively).

**Figure 3:**
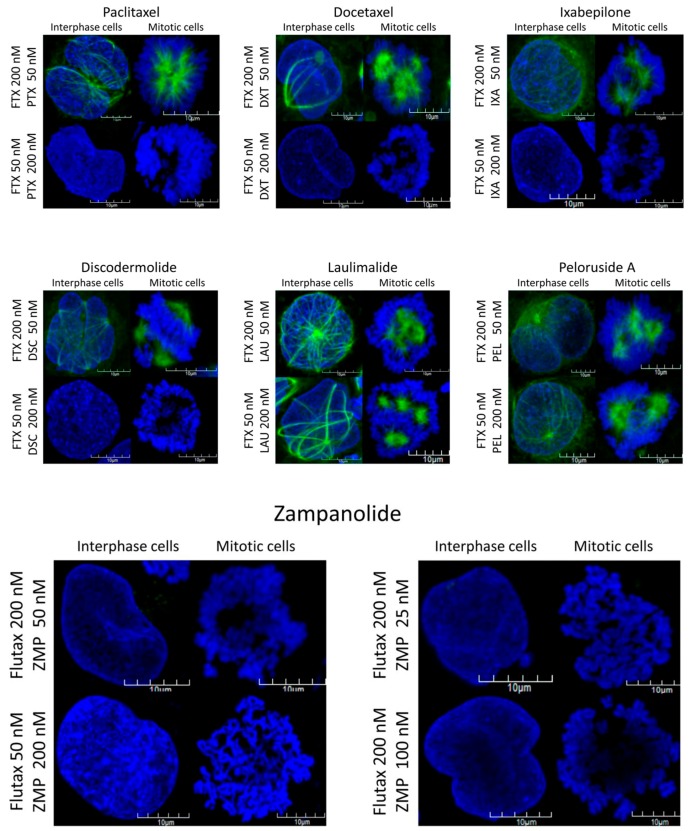
Competition for Flutax-2 binding of cellular microtubules by different microtubule-stabilizing agents.

ZMP has been shown by both Flutax competition experiments [2,39] and X-ray crystallography [15] to bind at the taxoid site, yet taxoid site amino acid mutations had little effect on its interactions with tubulin. We previously showed that a high concentration of PTX could compete for bound Flutax-2 but not at a low concentration, whereas because ZMP binds covalently to the taxoid site [2], both high and low concentrations of ZMP could displace the Flutax-2 [2,39] (Figure 3). Peloruside A, as expected, was unable to displace Flutax-2 because it binds at a distant, non-taxoid site on β-tubulin [16,40]. In the present study, we therefore tested other MSAs to see if they were effective in displacing Flutax and found that other taxoid site agents, including docetaxel, ixabepilone, and discodermolide, could displace Flutax similar to PTX, but laulimalide, similar to peloruside A, could not (Figure 3).

HL-60 human promyelocytic leukemic cells were co-treated with a combination of a microtubule-stabilizing agent (MSA) and Flutax-2 (FTX) for 16 h, stained with (4′,6-diamidino-2-phenylindole) (DAPI), and the fluorescence of the cells was examined in a confocal microscope. Taxoid site ligands, when in excess at 200 versus 50 nM FTX, inhibit FTX binding since most of the taxoid sites are occupied by the non-fluorescent taxoid site ligand, thus no green fluorescent microtubles (MTs) are seen. When FTX is in excess to the taxoid site ligands, the MTs fluoresce green since FTX occupies most of the binding sites on the MTs. Regardless of the concentration of laulimalide (LAU) or peloruside A (PEL), the MTs always fluoresce green since simultaneous binding of these two MSAs at the LAU/PEL site and Flutax-2 (FTX) at the taxoid site can occur. When zampanolide (ZMP) is in excess over FTX, no green fluorescence of the MT is apparent, as was seen with the other taxoid site ligands. In contrast, when FTX is in excess, no green fluorescence is present, even at a low concentration of ZMP (25 nM). This lack of green fluorescence when FTX is in excess indicates that ZMP can out-compete FTX because it covalently occupies the taxoid binding site, preventing FTX from displacing it. The PTX, PEL, and ZMP images have been previously published [39] and are shown with permission from the publisher Elsevier. Abbreviations are: PTX = paclitaxel; DTX = docetaxel; IXA = ixabepilone; DSC = discodermolide, and FTX = Flutax-2.

### 2.3. Lack of Synergistic Interactions between ZMP and Other MSAs

**Figure 4:**
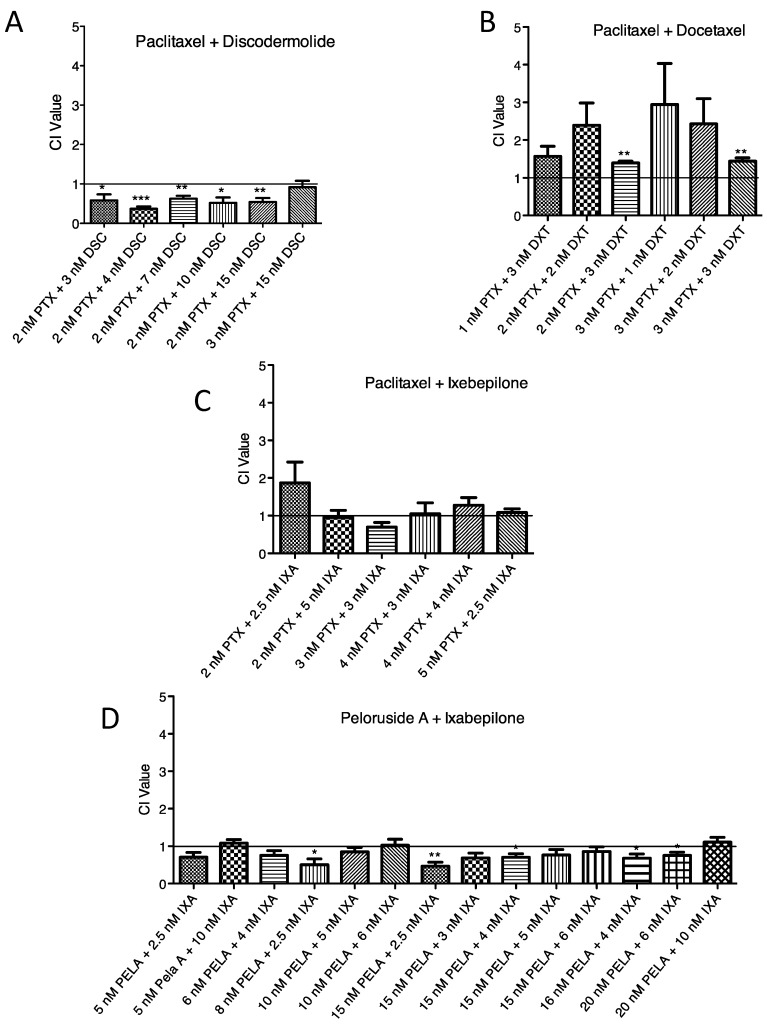
Synergistic interactions between MSAs. The Combination Index (CI) is graphed for combinations of MSAs in 1A9 cells given two at a time: (**A**) Paclitaxel (PTX) and discodermolide (DSC); (**B**) Paclitaxel and docetaxel (DXT); (**C**) Paclitaxel and ixabepilone (IXA), and (**D**) peloruside A (PELA) and ixabepilone are presented as the mean CI value ± SEM. * *p* < 0.05; ** *p* < 0.01; *** *p* < 0.001; one-sample Student’s *t*-test compared to 1.0. Values less than 1.0 indicate synergy between the two compounds; values greater than 1.0 indicate antagonism; values equal to 1.0 indicate additivity.

**Figure 5:**
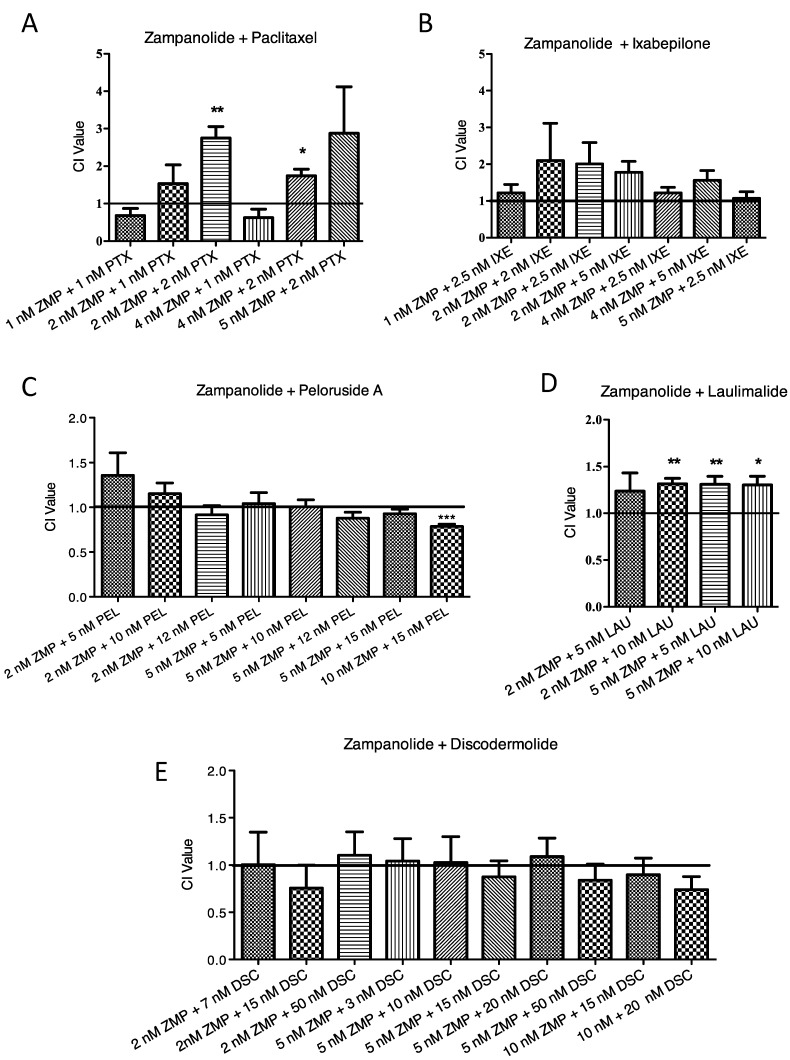
Synergistic interactions of ZMP with selected MSAs. The Combination Index (CI) is graphed for ZMP given in combination with other MSAs: (**A**) Paclitaxel (PTX); (**B**) Ixabepilone (IXE); (**C**) Peloruside A (PEL); (**D**) Laulimalide (LAU), and (**E**) discodermolide (DSC). Data are presented as the mean CI value ± SEM. * *p* < 0.05; ** *p* < 0.01; *** *p* < 0.001; one-sample Student’s *t*-test compared to 1.0. Values less than 1.0 indicate synergy between the two compounds; values greater than 1.0 indicate antagonism; values equal to 1.0 indicate additivity.

MSAs that bind to different sites on tubulin have been shown to synergize together if the appropriate concentrations of the two compounds are combined, as shown in Figure 4. CI values for Figure 4 are presented in Supplementary Data Table S2. For example, in 1A9 cells, peloruside A in combination with ixabepilone had a greater effect on cell growth than the sum of the two compounds. PTX, however, did not synergize with ixabepilone, because PTX and ixabepilone bind at the same site, the taxoid site. The exception to the rule is discodermolide, a taxoid site MSA that has previously been shown to synergize with PTX [20,21,22], despite being able to compete for Flutax binding (Figure 3). The 1A9 ovarian carcinoma cells were therefore treated with ZMP in combination with three other taxoid site MSAs and two laulimalide/peloruside site MSAs to determine if ZMP could synergize with either group of compounds (Figure 5). CI values for Figure 5 are presented in Supplementary Data Table S3. No synergy was seen between ZMP and either a taxoid site MSA or a laulimalide/peloruside site MSA. Some antagonism was observed between PTX and ZMP, and laulimalide and ZMP.

### 2.4. Zampanolide Effect on Cell Migration in Culture

**Figure 6:**
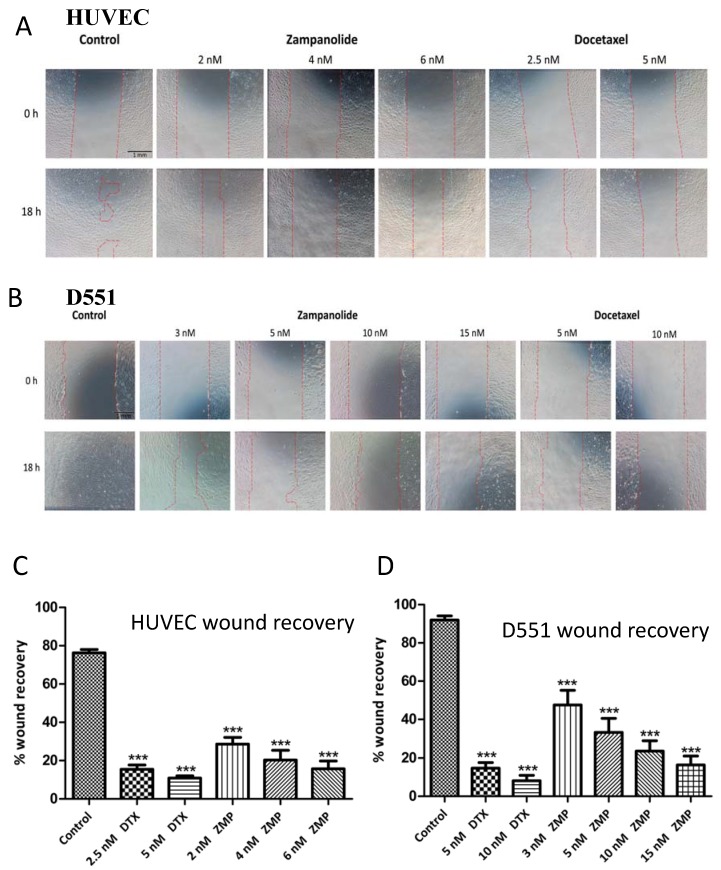
Zampanolide (ZMP) and docetaxel (DTX) inhibition of human umbilical vein endothelial cell HUVEC and D551 cell migration. (**A**) HUVEC cell images at 0 and 18 h; (**B**) D551 images at 0 and 18 h; (**C**) Summary of wound repair for HUVEC cells; (**D**) Summary of wound repair for D551 cells. (**A**/**B**) Representative images (4× magnification) of scratch wounds were taken at time 0 h (upper panel) and 18 h later (lower panel). The dotted lines indicate the approximate location of the edges of the wound at *t* = 0. Control cells showed almost complete closure of the wound by 18 h, whereas drug-treated cells still contained a wound, indicating that the rate of migration had been inhibited. (**C**/**D**) Quantification of wound inhibition by ZMP and docetaxel compared to the control without MSA treatment. Data are presented as the mean % wound recovery ± SEM (*n* = 6 independent experiments with two or three technical repeats per experiment). A Dunnett’s multiple comparison post-hoc test was used to determine the significance of the difference in drug treated samples with that of the control (*** *p* < 0.001).

To ascertain whether ZMP has the potential to prevent metastasis of cancer cells, its ability to inhibit cell migration in a wound scratch assay was carried out. The ability of human umbilical vein endothelial cells (HUVEC) or D551 human fibroblasts to repair a scratch made in the monolayer with a pipette tip was tested in the absence and presence of ZMP and docetaxel (Figure 6). Both compounds significantly inhibited wound recovery in both cell lines in a concentration-dependent manner. Docetaxel was slightly more potent than ZMP at inhibiting cell migration.

## 3. Discussion

### 3.1. ZMP Cytotoxicity and ZMP Activity in Resistant Cells

ZMP is a low nanomolar cytotoxic agent and was highly effective in every cell type tested in the present study. Its mode of action was determined in 2009 to be MT stabilization by Field et al. [1], and it was shown three years later to bind covalently to the amino acid residue His229 on β-tubulin and possibly also Asn228 [2]. Two other MSAs, cyclostreptin [3] and taccalonolide AJ [4], also bind β-tubulin covalently, although the exact amino acids involved with taccalonolide AJ are not known; however, the peptide segments involved are the same as for cyclostreptin, including Thr220 and Asn228. ZMP also may form a covalent bond with Asn228 in the taxoid site, although the evidence favors His229 [2]. As a potential anticancer agent for treating solid tumors of the breast, ovary, lung, prostate, kidney, and head and neck, this sensitivity of cells to ZMP that have acquired resistance to PTX, a major chemotherapeutic drug used in the clinic, is promising and indicates that further characterization of ZMP is desirable. It is not known whether ZMP is unable to act as a substrate for the efflux pump or whether it evades being pumped out of the cell by its formation of a covalent bond [5]. A number of novel MSAs that do not bind covalently are able to overcome the acquired resistance of cancer cells, either because they are not substrates for the P-gp drug efflux pump or because they bind with relatively equal affinity to all the main β-tubulin isotypes, including β-III tubulin that is known to confer resistance in some cell lines [41]. For example, taccalonolides are able to circumvent both mechanisms of resistance by their unique mechanism of MT stabilization, and interestingly, do not polymerize purified tubulin in vitro [4,42].

### 3.2. Action of ZMP on Cells with β-Tubulin Mutations

Mutations in the MSA binding sites of β-tubulin have been described that confer resistance on the compounds that bind those sites [9,10,11,12,13]. Some of these mutations occurred spontaneously in cell cultures exposed for extended periods of time to step-wise increases in MSA [9,10,11,12], whereas others occurred after mutagenesis of the cells followed by treatment with high concentrations of MSAs [7,13]. The random mutagenesis approach used by Yin et al. [8] identified a number of novel mutations but also brought up other mutations previously described in the literature. Some of these mutations have been observed in patients that have neuronal abnormalities suggesting that single amino acid changes may cause drug resistance as well as contribute to deficits in brain development, presumably as a result of the effect of the mutations on MT function in neuronal axons and dendrites [13].

Before electron crystallography physically confirmed the taxoid [15] and laulimalide/peloruside [16] binding sites, β-tubulin mutations conferring resistance provided a form of indirect biological evidence for the location of the binding pockets [43]. Schibler and Cabral [7] and Giannakakou et al. [9,10] were the first to describe PTX- and epothilone-resistant cell lines with mutations in the taxoid binding site. Surprisingly, in the present study, none of the taxoid site mutations affected the sensitivity of the cells to ZMP, despite its binding to the same site. The PTX-resistant cells showed some cross-resistance to ixabepilone, and the epothilone-resistant cell lines, particularly B10, showed cross-resistance to PTX. The mutations were located in amino acid residues 276 (A8) and 284 (B10), both located in the PTX binding pocket. Thr276, the amino acid mutated in A8 cells, can form two hydrogen bonds with ZMP [15], and it could therefore be predicted that these cells should be at least partially resistant to ZMP due to the loss of hydrogen bonding. Thus, even though ZMP forms two hydrogen bonds with this residue, either its covalent binding mechanism overcomes the mutation, faster than it does with B10 cells for example, or other viable hydrogen bonds can be formed with the mutated isoleucine. Since these hydrogen bonds are forming with the amino acid backbone of threonine and not the side chain, the same bonding opportunities should exist with isoleucine, providing the torsion angles of the amino acids are not significantly different between the wild type and mutant. This might explain why ZMP is fully active in the A8 cells. This model is further supported by the crystal structure of epothilone A [15], and it is not surprising that this is the amino acid that spontaneously mutated when the cells were exposed to high epothilone A concentrations, given its extensive interactions with epothilone A. Although epothilone A may be able to retain one of its hydrogen bonds with the backbone of isoleucine, its N20 hydrogen bond to the polar threonine side chain would be lost since the side chain of isoleucine is nonpolar and unable to form a hydrogen bond.

We previously showed that a high concentration of PTX could compete for bound Flutax, whereas a high concentration of peloruside A could not because it binds at a distant, non-taxoid site on β-tubulin [2,39,40]. In those studies, because ZMP binds covalently to the taxoid site, it was able to displace Flutax at high as well as low concentrations. In the present study, we therefore tested other MSAs to see if they were effective in displacing Flutax, and found, as expected, that other taxoid site agents such as docetaxel, ixabepilone, and discodermolide could displace Flutax, but laulimalide, which binds a different site, could not (Figure 3). These results, plus our earlier study [2,39], confirmed that ZMP binds to the taxoid site where Flutax binds, and does not bind at the laulimalide/peloruside site. It is unclear why cells mutated at the taxoid site show cross-resistance to both PTX and epothilone but are not resistant to ZMP, such as the B10 cell line. The explanation, however, presumably lies in the covalent binding nature of ZMP, as given enough time, ZMP would irreversibly bind to more and more of the taxoid binding sites on the MT, out-competing a reversible binding ligand. A number of other mutant cell lines have also been described that are resistant to some but not all of the PTX site compounds, indicating that ligands interact in different, unique ways with the taxoid pocket [13]. Binding site interactions have been reviewed by Field et al. [44]. Yin et al. [13] and Kanakkanthara et al. [43] provide brief overviews of the known mutations. The mutations all tend to cluster around the proposed binding sites; however, mutations outside the binding site can still affect the ability of an MSA to bind due to allosteric changes in β-tubulin. Tubulin mutations outside the recognized binding sites can cause changes in the stability of a MT, even in the absence of drugs. Similar to the mutational studies of the taxoid site, mutations have been described for the laulimalide/peloruside site [11,12] indicating the importance of amino acid residues 298 and 308 in the binding of these MSAs. Although there are no confirmed hypotheses on the mechanism of resistance to these drugs, it is assumed that reduced binding of the ligand (a decrease in *K*_m_) results in the decreased cytotoxicity (increased resistance), rather than a non-specific defect in MT function as a result of the mutation. This is supported by the fact that the MSAs are still able to cause the typical aberrations in MTs leading to cell cycle arrest, spindle defects, and multiple asters if the concentration of the MSA is increased high enough [11,45].

Although ZMP is a ligand of the taxoid site, it is not affected by mutations in the taxoid binding site in mutant 1A9 cells to the same extent that PTX and other taxoid site ligands are. The same was seen with cyclostreptin [3], indicating, as suggested by Singh et al. [5], that irreversible inhibitors would be effective against resistant mutants as they do not affect the extent of inhibition but only the rate at which the inhibited complex forms. Therefore, if sufficient exposure time was available, the reaction might be slower, but full inhibition would eventually occur due to the irreversible binding. Given this, it would be interesting to treat B10 cells (epothilone B mutants) with ZMP for an extended time to see if their resistance to ZMP (resistance ratio = 3.2) can be overcome by the longer incubation.

An attempt was made to create a ZMP-resistant cell line, and although no ZMP resistance was seen, the cells developed 11-fold resistance to PTX. The mutation in these cells, if one exists, is therefore likely to be located within the taxoid binding pocket. It is unlikely, however, that it is an isotype shift or a change in the general stability of tubulin since ixabepilone, peloruside, and laulimalide remained fully active in the cells. It has been shown that mutations can occur in a binding site but not all ligands that bind to that site are affected by the mutation. For example, epothilone B is fully active in PTX-resistant cell lines [9], and laulimalide in 1A9-R1 cells [11]. An interesting question is why ZMP, ixabepilone, laulilmalide, and peloruside A all showed resistance ratios of less than one (0.4 to 0.6) in the ZMP-conditioned cells. One possible explanation is that there may have been a mutation in the PTX binding site that affected PTX selectively but also had general allosteric effects on MT stability, making the MTs more stable and therefore increasing the sensitivity to the four other MSAs, regardless of whether they bound at the taxoid site or the laulimalide/peloruside site. An alternative possibility, however, is that the cell line was generally less healthy after the extended, intermittent ZMP exposures, making the cells more sensitive to any stressor, although not to PTX. Since the decreases in resistance ratios were small, a third possibility is that they represent natural biological variation in the measurements.

### 3.3. Lack of Synergy with ZMP

Combination therapy involves the administration of two drugs that either act synergistically with each other or are additive. This approach has become a common mechanism used clinically to improve the action profiles of therapeutic drugs and avoid the development of resistance. Synergistic interactions allow shorter administration times and lower drug doses, resulting in decreased side effect toxicity. Currently, the taxanes are used in the clinic in a number of different settings in combination with other drugs.

It has been shown that taxoid site MSAs can synergize with laulimalide or peloruside A both in vitro [46,47] and in cells [17,18,19]. This synergism is thought to be due to differences in the binding mode of the compounds and the mechanism by which they cause stabilization of the MTs. In addition, PTX and some of its derivatives can synergize with MT destabilizing agents, for example, PTX and vinorelbine [48], PTX and vinblastine when administered sequentially [49], and PTX with 2-methoxyestradiol both in vivo and in vitro [50,51]. Thus, if two compounds target tubulin at different binding sites they are more likely to synergize with one another. It seems unlikely that two drugs binding to the same site would synergize with one another; however, as mentioned earlier, PTX and discodermolide are an exception, as they synergize together in both cell culture [20,21,22] and xenograft tumor models [52]. It has been shown that the stabilization of MTs by PTX and discodermolide complement each other [23,24], and although both drugs bind the taxoid site, they have unique poses within this site and activate the M-loop differentially [24], allowing synergy to occur. Complementary interactions may also occur at both the interdimer and lateral interfaces of tubulin in addition to the binding pocket [53].

### 3.4. ZMP Effect on Cell Migration

Tumor cells have the potential to metastasize, and this leads to formation of new blood vessels in metastatic tumors [26] and secondary tumor foci at distant anatomic sites [25]. The angiogenesis process involves proliferation and migration of endothelial cells, with migration mediated by focal adhesions. Taxoid site ligands and laulimalide and peloruside A all have varying degrees of anti-angiogenic activities through inhibition of cell migration and interference with the formation of focal adhesions [31,32,33,34,35,36,37,54]. It is well known that MTs are important in the directional migration of endothelial cells [36,37], and thus it is not surprising that MTAs can inhibit this process. Using the wound scratch assay, ZMP was shown to be a potent inhibitor of cell migration, similar to docetaxel. No attempt was made to titrate the ZMP concentration to determine if the IC_50_ for the anti-migratory effect was lower than the IC_50_ for growth inhibition, as shown by Ganguly et al. in another study on the MSAs PTX and peloruside A [54]. The concentrations of ZMP needed to significantly inhibit the migration of HUVEC and D551 cells in the wound scratch assay were similar to the IC_50_ values for growth inhibition, and showed a highly dose-dependent effect, indicating that the migration inhibitory process was not saturated.

## 4. Materials and Methods

### 4.1. Compounds

ZMP and laulimalide were isolated and purified from the marine sponge *Cacospongia mycofijiensis* collected from ’Eua and Vava’u, Tonga [1]. Synthetic ZMP was prepared as described by Zurwerra et al. [38]. Synthetic discodermolide was prepared as described by Paterson et al. [55]. Peloruside A was isolated and purified from the marine sponge *Mycale hentscheli* collected from Pelorus Sound, New Zealand [56]. PTX was purchased from Sigma Chemical Co. (St. Louis, MO, USA), docetaxel was purchased from LC Laboratories^®^ Woburn, MA, USA and ixabepilone from Bristol-Myers Squibb (USA). Flutax-2 (7-*O*-[*N*–(2,7-difluoro-4′-fluoresceincarbonyl)-l-alanyl]-paclitaxel) was prepared by Wei Shuo Fan (Chinese Academy of Medical Sciences, Beijing, China) for Fernando Díaz, CIB, CSIC, Madrid, Spain. Stock compounds were stored in absolute ethanol or dimethyl sulfoxide (DMSO) at −80 °C.

### 4.2. Cell Lines, Cell Culture, and the MTT Cell Proliferation Assay

The parental (1A9) and β-tubulin mutant (PTX10, PTX22, A8, B10 and 1A9-L4) human ovarian carcinoma cell lines were a gift from Paraskevi Giannakakou, Weill Medical College of Cornell University, New York, NY, USA. The 1A9-R1 cells were prepared as described in Kanakkanthara et al. [11]. Human skin fibroblast cells (D551) were a kind gift from Darren Day, Victoria University of Wellington, New Zealand (ATCC^®^ CCL-110™). Human umbilical vein endothelial cells (HUVEC) were a kind gift from Darren Day and Melanie McConnell of Victoria University of Wellington, New Zealand.

Unless otherwise stated, cells were cultured at 37 °C in a 5% CO_2_/air atmosphere in RPMI-1640 medium (Invitrogen, Auckland, NZ, USA) supplemented with 10% fetal calf serum (Invitrogen), and 100 units/mL penicillin/streptomycin (Invitrogen). For 1A9 cells, 0.25 units/mL insulin (Sigma Chemical Co., St. Louis, MO, USA) was added to the medium. HUVEC cells were cultured in MCDB131 medium (Invitrogen) supplemented with 2% FCS, 10 ng/mL epidermal growth factor (Invitrogen), 12 μg/mL endothelial cell growth supplement (Sigma), 1 μg/mL hydrocortisone (Sigma), 10 U/mL heparin (Sigma), 2 mM l-glutamine (Sigma), and 100 U/mL penicillin/streptomycin. Adherent cells were detached from the surface using trypsin-EDTA (Invitrogen) and centrifuged for 5 min at 300× *g* to pellet the cells. The MTT (3-(4,5-dimethylthiazol-2-yl)-2,5-diphenyltetrazolium bromide) cell proliferation assay was carried out as previously described [57].

### 4.3. The Combination Index (CI Value) for Synergy Determinations in 1A9 Cells

Procedures for determining the CI value for combinations of two MSAs were as described by Wilmes et al. [19]. Basically, 1A9 cells were treated for 48 h with individual drugs to determine a concentration-response curve for choosing a combination of two drugs to test whether the drugs could synergize. A number of different combination concentrations were chosen for each drug pair (*n* = 4 or more independent experiments). Generally, the concentrations of drugs were chosen that led to about 50% inhibition of cell proliferation when added on their own to cells. The combination index (CI) equation [58,59] is widely used to quantitatively assess synergy: CI = (D_1_/Dx_1_ + D_2_/Dx_2_) in which Dx_1_ and Dx_2_ are the concentrations used in the combination dose, and D_1_ and D_2_ are the concentrations of each drug that would produce the same response as the combination dose if given individually (determined from the corresponding concentration-response curve). A CI value of 1 denotes additive effects; a value less than 1 denotes a synergistic interaction between the two drugs, and a value greater than 1 denotes an antagonistic relationship. For statistical comparisons, a one-sample Student’s *t*-test (GraphPad Prism v5.0, San Diego, CA, USA) was used to compare CI values to a set value of 1.0. Only CI values of ≤0.8 were considered to be biologically effective synergy. When synergy was found between a drug pair, repeat determinations were carried out to ensure a robust statistical CI value for that combination of compounds.

### 4.4. Wound Scratch Healing Assay with Human Umbilical Vein Endothelial Cells (HUVEC) and D551 Cells

To monitor cell migration, a wound scratch assay was used, as previously described by Chan et al. [33]. In brief, cells were plated at 5 × 10^4^ cells/well of a 24-well plate and grown for 2 days or until confluent. Cells were then treated with the drug for 5 h in their normal medium but lacking FCS or supplemented growth factors to starve the cells of pro-migratory and proliferative factors. A perspex plate insert [33] was used as a mechanical guide to create a consistent scratch in each well with a 200 μL pipette tip. The cells were then washed 3 times in warm PBS (pH 7.4), and complete medium containing the drug, FCS, and growth factors was added to the wells at *t* = 0. Photos of the cells were taken at 0 and 18 h with an Olympus IX51 inverted microscope using the 4× objective lens. Both photos were taken of the same area using marker lines drawn on the bottom of the well as a guide to orientate the images. The area of the wound was then calculated using the open resource tool of ImageJ (NIH, Baltimore, MD, USA). The extent of wound healing (taken as a measure of cell migration) was determined by measuring the area of the open wound at both 0 and 18 h and calculating the percentage of wound closure after 18 h. Statistical comparisons were made using a one-way ANOVA, with the drug-treated samples compared to the control using a Dunnett’s multiple comparison post-hoc test (GraphPad Prism v5.0).

## 5. Conclusions

ZMP, first discovered in a sponge extract by Tanaka and Higa [60] in 1996 and later identified as an MSA in 2009 by Field et al. [1], is a covalent binding agent with potent, low nanomolar activity. It is not susceptible to mutations in single amino acids in the taxoid site, despite the evidence that it selectively binds to the taxoid site on β-tubulin. As expected, ZMP did not synergize with other taxoid site MSAs, but surprisingly also did not synergize with laulimalide/peloruside site MSAs. ZMP was an effective inhibitor of cancer cell migration in culture. These properties suggest that ZMP would be of interest to investigate in preclinical animal models to determine its effectiveness in vivo at preventing tumor growth and metastasis.

## Figures and Tables

**Table 1:** IC_50_ values for microtubule-stabilizing agents in 1A9 cells.

Compound	IC_50_ ± SEM (nM)
Taxoid site ligands	
Zampanolide	9.54 ± 0.85
Paclitaxel	3.71 ± 0.30
Docetaxel	3.55 ± 0.43
Ixabepilone	6.65 ± 0.33
Discodermolide	138 ± 12
Laulimalide/Peloruside site ligands	
Peloruside A	23.3 ± 1.1
Laulimalide	9.71 ± 0.28

IC_50_ values in 1A9 cells (mean ± SEM) after 48 h of drug treatment are presented (*n* = the number of independent biological replicates).

**Table 2:** Cytotoxicity of zampanolide (ZMP) in different cell lines.

Cell Line	Source of ZMP	IC_50_ ± SEM (nM)	Duration (h)
1A9	natural	8.2 ± 0.1	72
1A9	synthetic	4.6 ± 1.3	72
1A9 [1]	natural	14.3 ± 2.4	72
HL-60 [1]	natural	4.3 ± 1.1	48
D551	synthetic	7.3 ± 1.2	72
HUVEC	synthetic	0.6 ± 0.1	72
HUVEC	synthetic	1.0 ± 0.4	120

IC_50_ values of zampanolide in different cell lines determined using the MTT (3-(4,5-dimethylthiazol-2-yl)-2,5-diphenyltetrazolium bromide) cell proliferation assay. Duration is the time in which each cell line was treated with zampanolide before MTT was added; *n* is the number of independent biological replicates.

**Table 3:** IC_50_ values for MSAs in 1A9 parental cells and β-tubulin mutant cell lines.

Cell Line	Resistance to	Paclitaxel	Ixabepilone	Zampanolide	Peloruside A	Laulimalide
1A9		4.2 ± 0.3	7.3 ± 0.6	8.2 ± 1.0	20.1 ± 0.9	8.3 ± 0.5
PTX10	PTX and EPO	91.7 ±8.2	54.9 ± 9.6	2.3 ± 0.9	17.5 ± 1.2	11.0 ± 1.0
PTX22	PTX	100 ± 14.1	11.4 ± 1.7	9.2 ± 3.9	19.4 ± 5.4	10.6 ± 2.7
A8	EPO and PTX	94.4 ± 5.6	99.8 ± 0.6	14.9 ± 4.6	14.0 ± 2.4	7.2 ± 1.1
B10	EPO	17.2 ± 4.3	106 ± 6.5	8.6 ± 3.2	24.9 ± 1.9	10.8 ± 1.0
1A9-R1	PLA	8.8 ± 2.5	14.7 ± 3.3	5.9 ± 1.6	90.9 ± 8.5	9.8 ± 1.5
1A9-L4	LAU and PLA	4.2 ± 0.1	4.4 ± 0.4	4.7 ± 1.0	351 ± 126	344 ± 150

The average 72 h IC_50_ values of different MSAs in the parental 1A9 cell line and cloned mutant 1A9 cell lines are presented as the mean IC_50_ value ± SEM (*n* = 3 or more biological replicates). The specific mutations for each cell line are: PTX10 Phe272Val; PTX22 Ala374Thr; A8 Thr276Ile; B10 Arg284Gln; 1A9-R1 Ala298Thr; 1A9-L4 Arg308His(70%)/Cys(30%). Resistance ratios are presented in Figure 2 and Supplementary Data Table S1. PTX = paclitaxel, EPO = epothilone, PLA = peloruside A, and LAU = laulimalide.

## Supplementary Materials

Supplementary materials can be found at www.mdpi.com/1422-0067/18/5/971/s1.

## Abbreviations

CI	Combination index
MT	Microtubule
MTA	Microtubule-targeting agent
MSA	Microtubule-stabilizing agent
PTX	Paclitaxel
P-gp	*p*-glycoprotein
ZMP	Zampanolide

## References

[1] Field J.J., Singh A.J., Kanakkanthara A., Halafihi T., Northcote P.T., Miller J.H. (2009). Microtubule-stabilizing activity of zampanolide, a potent macrolide isolated from the Tongan marine sponge *Cacospongia mycofijiensis*. J. Med. Chem..

[2] Field J.J., Pera B., Calvo E., Canales A., Zurwerra D., Trigili C., Rodríguez-Salarichs J., Matesanz R., Kanakkanthara A., Wakefield S.J. (2012). Zampanolide, a potent new microtubule-stabilizing agent, covalently reacts with the taxane luminal site in tubulin α,β-heterodimers and microtubules. Chem. Biol..

[3] Buey R.M., Calvo E., Barasoain I., Pineda O., Edler M.C., Matesanz R., Cerezo G., Vanderwal C.D., Day B.W., Sorensen E.J. (2007). Cyclostreptin binds covalently to microtubule pores and lumenal taxoid binding sites. Nat. Chem. Biol..

[4] Risinger A.L., Li J., Bennett M.J., Rohena C.C., Schriemer D.C., Mooberry S.L. (2013). Taccalonolide binding to tubulin imparts microtubule stability and potent in vivo activity. Cancer Res..

[5] Singh J., Petter R.C., Baillie T.A., Whitty A. (2011). The resurgence of covalent drugs. Nat. Rev. Drug Discov..

[6] Cabral F., Abraham I., Gottesman M.M. (1981). Isolation of a taxol-resistant Chinese hamster ovary cell mutant that has an alteration in α-tubulin. Proc. Natl. Acad. Sci. USA.

[7] Schibler M.J., Cabral F. (1986). Taxol-dependent mutants of Chinese hamster ovary cells with alterations in α- and β-tubulin. J. Cell Biol..

[8] Yin S., Bhattacharya R., Cabral F. (2010). Human mutations that confer paclitaxel resistance. Mol. Cancer Ther..

[9] Giannakakou P., Sackett D.L., Kang Y.K., Zhan Z., Buters J.T., Fojo T., Poruchynsky M.S. (1997). Paclitaxel-resistant human ovarian cancer cells have mutant β-tubulins that exhibit impaired paclitaxel-driven polymerization. J. Biol. Chem..

[10] Giannakakou P., Gussio R., Nogales E., Downing K.H., Zaharevitz D., Bollbuck B., Poy G., Sackett D., Nicolaou K.C., Fojo T. (2000). A common pharmacophore for epothilone and taxanes: Molecular basis for drug resistance conferred by tubulin mutations in human cancer cells. Proc. Natl. Acad. Sci. USA.

[11] Kanakkanthara A., Wilmes A., O'Brate A., Escuin D., Chan A., Gjyrezi A., Crawford J., Rawson P., Kivell B., Northcote P.T. (2011). Peloruside- and laulimalide-resistant human ovarian carcinoma cells have βI-tubulin mutations and altered expression of βII- and βIII-tubulin isotypes. Mol. Cancer Ther..

[12] Begaye A., Trostel S., Zhao Z., Taylor R.E., Schriemer D.C., Sackett D.L. (2011). Mutations in the β-tubulin binding site for peloruside A confer resistance by targeting a cleft significant in side chain binding. Cell Cycle.

[13] Yin S.H., Zeng C.Q., Hari M., Cabral F. (2012). Random mutagenesis of β-tubulin defines a set of dispersed mutations that confer paclitaxel resistance. Pharm. Res..

[14] Basciano P.A., Matakas J., Pecci A., Civaschi E., Cagioni C., Bompiani N., Burger P., Christos P., Snyder J.P., Bussel J. (2015). β-1 tubulin R307H SNP alters microtubule dynamics and affects severity of a hereditary thrombocytopenia. J. Thromb. Haemost..

[15] Prota A.E., Bargsten K., Zurwerra D., Field J.J., Díaz J.F., Altmann K.-H., Steinmetz M.O. (2013). Molecular mechanism of action of microtubule-stabilizing anticancer agents. Science.

[16] Prota A.E., Bargsten K., Northcote P.T., Marsh M., Altmann K.-H., Miller J.H., Díaz J.F., Steinmetz M.O. (2014). Structural basis of microtubule stabilization by laulimalide and peloruside A. Angew. Chem. Int. Ed. Engl..

[17] Clark E.A., Hills P.M., Davidson B.S., Wender P.A., Mooberry S.L. (2006). Laulimalide and synthetic laulimalide analogues are synergistic with paclitaxel and 2-methoxyestradiol. Mol. Pharm..

[18] Wilmes A., Bargh K., Kelly C., Northcote P.T., Miller J.H. (2007). Peloruside A synergizes with other microtubule stabilizing agents in cultured cancer cell lines. Mol. Pharm..

[19] Wilmes A., O’Sullivan D., Chan A., Chandrahasen C., Paterson I., Northcote P.T., La Flamme A.C., Miller J.H. (2011). Synergistic interactions between peloruside A and other microtubule-stabilizing and destabilizing agents in cultured human ovarian carcinoma cells and murine T cells. Cancer Chemother. Pharmacol..

[20] Giannakakou P., Fojo T. (2000). Discodermolide: Just another microtubule-stabilizing agent? No! A lesson in synergy. Clin. Cancer Res..

[21] Martello L.A., McDaid H.M., Regl D.L., Yang C.P., Meng D., Pettus T.R., Kaufman M.D., Arimoto H., Danishefsky S.J., Smith A.B. (2000). Taxol and discodermolide represent a synergistic drug combination in human carcinoma cell lines. Clin. Cancer Res..

[22] Honore S., Kamath K., Braguer D., Horwitz S.B., Wilson L., Briand C., Jordan M.A. (2004). Synergistic suppression of microtubule dynamics by discodermolide and paclitaxel in non-small cell lung carcinoma cells. Cancer Res..

[23] Khrapunovich-Baine M., Menon V., Verdier-Pinard P., Smith A.B., Angeletti R.H., Fiser A., Horwitz S.B., Xiao H. (2009). Distinct pose of discodermolide in taxol binding pocket drives a complementary mode of microtubule stabilization. Biochemistry.

[24] Prota A.E., Bargsten K., Redondo M., Smith A.B., Yang C.H., McDaid H.M., Paterson I., Horwitz S.B., Díaz J.F., Steinmetz M.O. (2017). Structural basis of microtubule stabilization by discodermolide. ChemBioChem.

[25] Brooks S.A., Lomax-Browne H.J., Carter T.M., Kinch C.E., Hall D.M.S. (2010). Molecular interactions in cancer cell metastasis. Acta Histochem..

[26] Tozer G.M., Kanthou C., Baguley B.C. (2005). Disrupting tumour blood vessels. Nat. Rev. Cancer.

[27] Liang C.C., Park A.Y., Guan J.L. (2007). In vitro scratch assay: A convenient and inexpensive method for analysis of cell migration in vitro. Nat. Protoc..

[28] Falasca M., Raimondi C., Maffucci T. (2011). Boyden chamber. Methods Mol. Biol..

[29] Hall D.M., Brooks S.A. (2014). In vitro invasion assay using Matrigel™: A reconstituted basement membrane preparation. Methods Mol. Biol..

[30] Belotti D., Vergani V., Drudis T., Borsotti P., Pitelli M.R., Viale G., Giavazzi R., Taraboletti G. (1996). The microtubule-affecting drug paclitaxel has antiangiogenic activity. Clin. Cancer Res..

[31] Lu H., Murtagh J., Schwartz E.L. (2006). The microtubule binding drug laulimalide inhibits vascular endothelial growth factor-induced human endothelial cell migration and is synergistic when combined with docetaxel (Taxotere). Mol. Pharmacol..

[32] Yang H., Ganguly A., Cabral F. (2010). Inhibition of cell migration and cell division correlates with distinct effects of microtubule inhibiting drugs. J. Biol. Chem..

[33] Chan A., Singh A.J., Northcote P.T., Miller J.H. (2015). Inhibition of human vascular endothelial cell migration and capillary-like tube formation by the microtubule-stabilizing agent peloruside A. Investig. New Drugs.

[34] Kamath K., Smiyun G., Wilson L., Jordan M.A. (2014). Mechanisms of inhibition of endothelial cell migration by taxanes. Cytoskeleton.

[35] Kaverina I., Straube A. (2011). Regulation of cell migration by dynamic microtubules. Semin. Cell. Dev. Biol..

[36] Ganguly A., Yang H., Sharma R., Patel K.D., Cabral F. (2012). The role of microtubules and their dynamics in cell migration. J. Biol. Chem..

[37] Ganguly A., Yang H., Zhang H., Cabral F., Patel K.D. (2013). Microtubule dynamics control tail retraction in migrating vascular endothelial cells. Mol. Cancer Ther..

[38] Zurwerra D., Glaus F., Betschart L., Schuster J., Gertsch J., Ganci W., Altmann K.-H. (2012). Total synthesis of (−)-zampanolide and structure-activity relationship studies on (−)-dactylolide derivatives. Chemistry.

[39] Field J.J., Calvo E., Northcote P.T., Miller J.H., Altmann K.-H., Díaz J.F., Wilson L., Correia J.J. (2013). Methods for studying microtubule binding site interactions: Zampanolide as a covalent binding agent. Methods in Cell Biology, Microtubules in Vitro.

[40] Gaitanos T.N., Buey R.M., Díaz J.F., Northcote P.T., Teesdale-Spittle P., Andreu J.M., Miller J.H. (2004). Peloruside A does not bind to the taxoid site on β-tubulin and retains its activity in multidrug-resistant cell lines. Cancer Res..

[41] Dumontet C., Jordan M.A. (2010). Microtubule-binding agents: A dynamic field of cancer therapeutics. Nat. Rev. Drug Discov..

[42] Risinger A.L., Jackson E.M., Polin L.A., Helms G.L., LeBoeuf D.A., Joe P.A., Hopper-Borge E., Ludueña R.F., Kruh G.D., Mooberry S.L. (2008). The taccalonolides: Microtubule stabilizers that circumvent clinically relevant taxane resistance mechanisms. Cancer Res..

[43] Kanakkanthara A., Teesdale-Spittle P.H., Miller J.H. (2013). Cytoskeletal alterations that confer resistance to anti-tubulin chemotherapeutics. Anticancer Agents Med. Chem..

[44] Field J.J., Díaz J.F., Miller J.H. (2013). The binding sites of microtubule-stabilizing agents. Chem. Biol..

[45] Kanakkanthara A., Eras J., Northcote P.T., Cabral F., Miller J.H. (2014). Resistance to peloruside A and laulimalide: Functional significance of acquired βI-tubulin mutations at sites important for drug-tubulin binding. Curr. Cancer Drug Targets..

[46] Gapud E.J., Bai R., Ghosh A.K., Hamel E. (2004). Laulimalide and paclitaxel: A comparison of their effects on tubulin assembly and their synergistic action when present simultaneously. Mol. Pharmacol..

[47] Hamel E., Day B.W., Miller J.H., Jung M.K., Northcote P.T., Ghosh A.K., Curran D.P., Cushman M., Nicolaou K.C., Paterson I. (2006). Synergistic effects of peloruside A and laulimalide with taxoid site drugs, but not with each other, on tubulin assembly. Mol. Pharmacol..

[48] Photiou A., Shah P., Leong L.K., Moss J., Retsas S. (1997). In vitro synergy of paclitaxel (Taxol) and vinorelbine (navelbine) against human melanoma cell lines. Eur. J. Cancer.

[49] Giannakakou P., Villalba L., Li H., Poruchynsky M., Fojo T. (1998). Combinations of paclitaxel and vinblastine and their effects on tubulin polymerization and cellular cytotoxicity: Characterization of a synergistic schedule. Int. J. Cancer.

[50] Ricker J.L., Chen Z., Yang X.P., Pribluda V.S., Swartz G.M., van Waes C. (2004). 2-methoxyestradiol inhibits hypoxia-inducible factor 1α, tumor growth, and angiogenesis and augments paclitaxel efficacy in head and neck squamous cell carcinoma. Clin. Cancer Res..

[51] Han G.Z., Liu Z.J., Shimoi K., Zhu B.T. (2005). Synergism between the anticancer actions of 2-methoxyestradiol and microtubule-disrupting agents in human breast cancer. Cancer Res..

[52] Huang G.S., Lopez-Barcons L., Freeze B.S., Smith A.B., Goldberg G.L., Horwitz S.B., McDaid H.M. (2006). Potentiation of taxol efficacy by discodermolide in ovarian carcinoma xenograft-bearing mice. Clin. Cancer Res..

[53] Khrapunovich-Baine M., Menon V., Yang C.P., Northcote P.T., Miller J.H., Angeletti R.H., Fiser A., Horwitz S.B., Xiao H. (2011). Hallmarks of molecular action of microtubule stabilizing agents: Effects of epothilone B, ixabepilone, peloruside A, and laulimalide on microtubule conformation. J. Biol. Chem..

[54] Ganguly A., Cabral F., Yang H., Patel K. (2015). Peloruside A is a microtubule-stabilizing agent with exceptional anti-migratory properties in human endothelial cells. Oncoscience.

[55] Paterson I., Florence G.J., Gerlach K., Scott J.P. (2000). Total synthesis of the antimicrotubule agent (+)-discodermolide using boron-mediated aldol reactions of chiral ketones. Angew. Chem. Int. Ed. Engl..

[56] West L.M., Northcote P.T., Battershill C.N. (2000). Peloruside A: A potent cytotoxic macrolide isolated from the New Zealand marine sponge *Mycale* sp.. J. Org. Chem..

[57] Hood K.A., West L.M., Northcote P.T., Berridge M.V., Miller J.H. (2001). Induction of apoptosis by the marine sponge (*Mycale*) metabolites, mycalamide A and pateamine. Apoptosis.

[58] Chou T.C., Talalay P. (1984). Quantitative analysis of dose-effect relationships: The combined effects of multiple drugs or enzyme inhibitors. Adv. Enzyme Regul..

[59] Berenbaum M.C. (1985). The expected effect of a combination of agents: The general solution. J. Theor. Biol..

[60] Tanaka J., Higa T. (1996). Zampanolide, a new cytotoxic macrolide from a marine sponge. Tetrahedron Lett..

