# Supplementary material for: Zampanolide, a Microtubule-Stabilizing Agent, Is Active in Resistant Cancer Cells and Inhibits Cell Migration
Source: Int J Mol Sci. 2017 May 3;18(5):971. doi: 10.3390/ijms18050971 (PMC5454884; doi:10.3390/ijms18050971)
Supplement: Supplementary file 1 [file ijms-18-00971-s001.pdf]

Zampanolide, a microtubule-stabilizing agent, is active in resistant cancer cells and inhibits cell migration.

Jessica J. Field, Peter T. Northcote, Ian Paterson, Karl-Heinz Altmann, J. Fernando Díaz, and John H. Miller

Supplementary Data

Table S1. IC_{50} values and resistance ratios for MSAs in 1A9 parental cells and tubulin mutant cell lines.

Cell line	Resistance to	Paclitaxel	Ixabepilone	Zampanolide	Peloruside A	Laulimalide
1A9	not resistant	4.2 ± 0.3	7.3 ± 0.6	8.2 ± 1.0	20.1 ± 0.9	8.3 ± 0.5
PTX10	PTX & EPO	91.7 ±8.2	54.9 ± 9.6	2.3 ± 0.9	17.5 ± 1.2	11.0 ± 1.0
**Resist Ratio (*p*)**		**22 ± 3 (0.002)**	**10 ± 1 (0.001)**	**0.8 ± 0.2** (ns)	**1.0 ± 0.02** (ns)	**1.4 ± 0.3** (ns)
PTX22	PTX	100 ± 14.1	11.4 ± 1.7	9.2 ± 3.9	19.4 ± 5.4	10.6 ± 2.7
**Resist Ratio (*p*)**		**24 ± 4 (0.005)**	**2.3 ± 0.4 (0.05)**	**2.4 ± 0.2** (ns)	**1.1 ± 0.3** (ns)	**1.2 ± 0.2** (ns)
A8	EPO & PTX	94.4 ± 5.6	99.8 ± 0.6	14.9 ± 4.6	14.0 ± 2.4	7.2 ± 1.1
**Resist Ratio (*p*)**		**4.7 ± 0.5 (0.002)**	**19 ± 3 (0.003)**	**0.6 ± 0.2** (ns)	**1.1 ± 0.1** (ns)	**1.1 ± 0.2** (ns)
B10	EPO	17.2 ± 4.3	106 ± 6.5	8.6 ± 3.2	24.9 ± 1.9	10.8 ± 1.0
**Resist Ratio (*p*)**		**34 ± 7 (0.02)**	**20 ± 3 (0.006)**	**3.2 ± 0.6 (0.02)**	**1.0 ± 0.3** (ns)	**0.9 ± 0.2** (ns)
1A9-R1	PLA	8.8 ± 2.5	14.7 ± 3.3	5.9 ± 1.6	90.9 ± 8.5	9.8 ± 1.5
**Resist Ratio (*p*)**		**1.8 ± 0.5** (ns)	**1.6 ± 0.5** (ns)	**0.6 ± 0.1 (0.003)**	**4.7 ± 0.3 (0.002)**	**1.1 ± 0.1** (ns)
1A9-L4	LAU & PLA	4.2 ± 0.1	4.4 ± 0.4	4.7 ± 1.0	351 ± 126	344 ± 150
**Resist Ratio (*p*)**		**1.3 ± 0.2** (ns)	**0.6 ± 0.1** (ns)	**0.8 ± 0.2** (ns)	**17 ± 7 (0.05)**	**50 ± 19 (0.05)**

The average 72 h IC_{50} values of different MSAs in the parental 1A9 cell line and cloned mutant cell lines are presented as the mean IC_{50} value ± SEM (n = 3 or more biological replicates). The resistance ratio (**Resist Ratio**) of the mutant cell line relative to the parental 1A9 cell line is presented in bold (mean ± SEM). Resist Ratio *p*-values are from a one-sample *t*-test. A resistance ratio of 1.0 demonstrates no difference in the IC50 value between the two cell lines; a value below one shows that the mutant cell line is more sensitive to the MSA; a value greater than one indicates that the mutant cell line is resistant to the compound. Some of the IC_{50} values for LAU treatment of 1A9-L4 cells were obtained from Dr Arun Kanakkanthara.

Table S2. CI values for different MSA combinations in 1A9 cells

**Paclitaxel**	**Discodermolide**	**CI ± SEM**	***n***	**p-value**
2 nM	3 nM	0.58 ± 0.15	6	**0.0454**
2 nM	4 nM	0.37 ± 0.06	6	**0.0002**
2 nM	7 nM	0.63 ± 0.07	6	**0.0039**
2 nM	10 nM	0.52 ± 0.14	6	**0.0184**
2 nM	15 nM	0.54 ± 0.10	6	**0.0064**
3 nM	15 nM	0.92 ± 0.16	6	ns
**Peloruside A**	**Ixabepilone**	**CI ± SEM**	***n***	**p-value**
5 nM	2.5 nM	0.71 ± 0.12	5	ns
5 nM	10 nM	1.08 ± 0.09	4	ns
6 nM	4 nM	0.75 ± 0.13	5	ns
8 nM	2.5 nM	0.50 ± 0.15	5	**0.0327**
10 nM	5 nM	0.85 ± 0.12	6	ns
10 nM	6 nM	1.03 ± 0.16	6	ns
15 nM	2.5 nM	0.46 ± 0.11	5	**0.0082**
15 nM	3 nM	0.69 ± 0.13	5	ns
15 nM	4 nM	0.70 ± 0.09	6	**0.0252**
15 nM	5 nM	0.76 ± 0.15	5	ns
15 nM	6 nM	0.86 ± 0.12	5	ns
16 nM	4 nM	0.68 ± 0.12	5	**0.05**
20 nM	6 nM	0.75 ± 0.09	6	**0.0378**
20 nM	10 nM	1.11 ± 0.12	5	ns
**Paclitaxel**	**Docetaxel**	**CI ± SEM**	***n***	**p-value**
1 nM	3 nM	1.57 ± 0.27	5	ns
2 nM	2 nM	2.39 ± 0.59	5	ns
2 nM	3 nM	1.40 ± 0.05	4	0.0035
3 nM	1 nM	2.95 ± 1.09	6	ns
3 nM	2 nM	2.43 ± 0.67	6	ns
3 nM	3 nM	1.44 ± 0.88	6	0.0039
**Paclitaxel**	**Ixabepilone**	**CI ± SEM**	***n***	**p-value**
2 nM	2.5 nM	1.87 ± 0.56	6	ns
2 nM	5 nM	0.95 ± 0.18	5	ns
3 nM	3 nM	0.70 ± 0.13	6	ns
4 nM	3 nM	1.05 ± 0.29	5	ns
4 nM	4 nM	1.27 ± 0.21	5	ns
5 nM	2.5 nM	1.08 ± 0.10	5	ns

Table S3. CI values for combinations of zampanolide + MSAs in 1A9 cells

**Zampanolide**	**Paclitaxel**	**CI ± SEM**	***n***	**p-value**
1 nM	1 nM	0.68 ± 0.19	4	ns
2 nM	1 nM	1.54 ± 0.50	4	ns
2 nM	2 nM	2.75 ± 0.30	4	0.0102
4 nM	1 nM	0.63 ± 0.23	4	ns
4 nM	2 nM	1.74 ± 0.17	4	0.0238
5 nM	2 nM	2.88 ± 1.24	4	ns
**Zampanolide**	**Ixabepilone**	**CI ± SEM**	***n***	**p-value**
1 nM	2.5 nM	1.22 ± 0.23	4	ns
2 nM	2 nM	2.05 ± 0.79	5	ns
2 nM	2.5 nM	2.01 ± 0.58	5	ns
2 nM	5 nM	1.78 ± 0.30	5	ns
4 nM	2.5 nM	1.22 ± 0.15	7	ns
4 nM	5 nM	1.56 ± 0.27	7	ns
5 nM	2.5 nM	1.07 ± 0.18	7	ns
**Zampanolide**	**Discodermolide**	**CI ± SEM**	***n***	**p-value**
2 nM	7 nM	1.00 ± 0.34	5	ns
2 nM	15 nM	0.76 ± 0.24	5	ns
2nM	50 nM	1.10 ± 0.24	5	ns
5 nM	3 nM	1.04 ± 0.23	5	ns
5 nM	10 nM	1.03 ± 0.27	5	ns
5 nM	15 nM	0.88 ± 0.17	5	ns
5 nM	20 nM	1.09 ± 0.19	5	ns
5 nM	50 nM	0.84 ± 0.17	5	ns
10 nM	15 nM	0.90 ± 0.17	5	ns
10 nM	20 nM	0.74 ± 0.14	5	ns
**Zampanolide**	**Peloruside A**	**CI ± SEM**	***n***	**p-value**
2 nM	5 nM	1.36 ± 0.25	4	ns
2 nM	10 nM	1.15 ± 0.12	7	ns
2 nM	12 nM	0.92 ± 0.10	4	ns
5 nM	5 nM	1.04 ± 0.12	4	ns
5 nM	10 nM	1.01 ± 0.08	8	ns
5 nM	12 nM	0.88 ± 0.07	4	ns
5 nM	15 nM	0.93 ± 0.05	8	ns
10 nM	15 nM	0.79 ± 0.03	7	**0.0002**
**Zampanolide**	**Laulimalide**	**CI ± SEM**	**n**	**p-value**
2 nM	5 nM	1.24 ± 0.20	4	ns
2 nM	10 nM	1.32 ± 0.06	8	0.001
5 nM	5 nM	1.31 ± 0.09	7	0.0109
5 nM	10 nM	1.30 ± 0.09	8	0.0143
